# Supplementary material for: Influence of Mutations of Conserved Arginines on Neuropeptide Binding in the DPP III Active Site
Source: Molecules. 2023 Feb 19;28(4):1976. doi: 10.3390/molecules28041976 (PMC9967118; doi:10.3390/molecules28041976)
Supplement: Supplementary file 1 [file molecules-28-01976-s001.zip › molecules-2219431-supplementary.pdf]

Supporting Information

Influence of mutations of conserved arginines on neuropeptide binding in the DPP III active site

Antonija Tomić, Zrinka Karačić, Sanja Tomić

Table of Content

1. Supplementary scheme 2

2. Supplementary figures 3

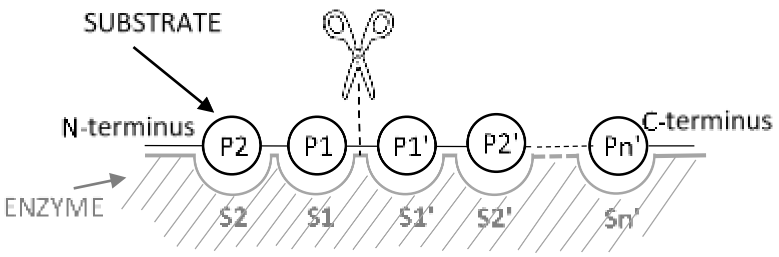

Scheme S1. Schematic representation of substrate binding to peptidase. The amino acid residues of the peptide substrate (designated as P1 to P2 and P1’ to Pn’ counting from the scissile peptide bond towards the N- and C-termini of the peptides, respectively) interacts with corresponding enzyme subsites (designated as S1 to S2 and S1’ and Sn’ counting from the scissile peptide bond toward the N-and C-termini of the enzyme, respectively).

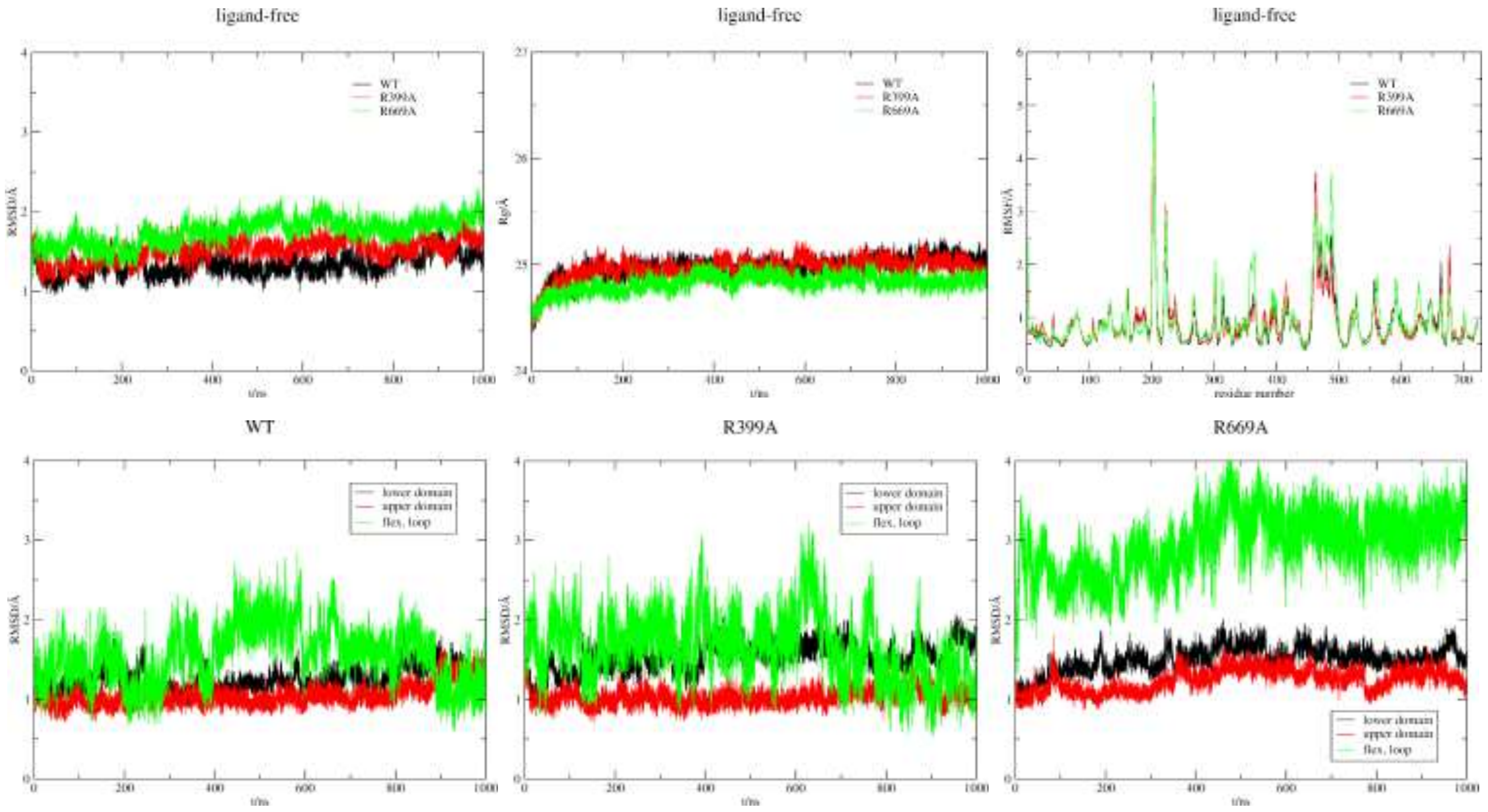

Figure S1. Profiles of root mean square deviations (RMSD), radius of gyration (Rg), and root mean square fluctuations (RMSF) from 1 µs long MD simulations of WT and mutated (R399A and R669A) ligand-free DPP III. Enzyme backbone atoms (Cα, C and N) were included in the analysis. RMSD and Rg profiles of the whole protein were calculated excluding the atoms from the flexible loop (residues 463-489). Amino acid residues 1-336, 375-421, and 669-726 are part of the lower protein domain, while residues 337-374, 422-462, and 490-668 are part of the upper protein domain.

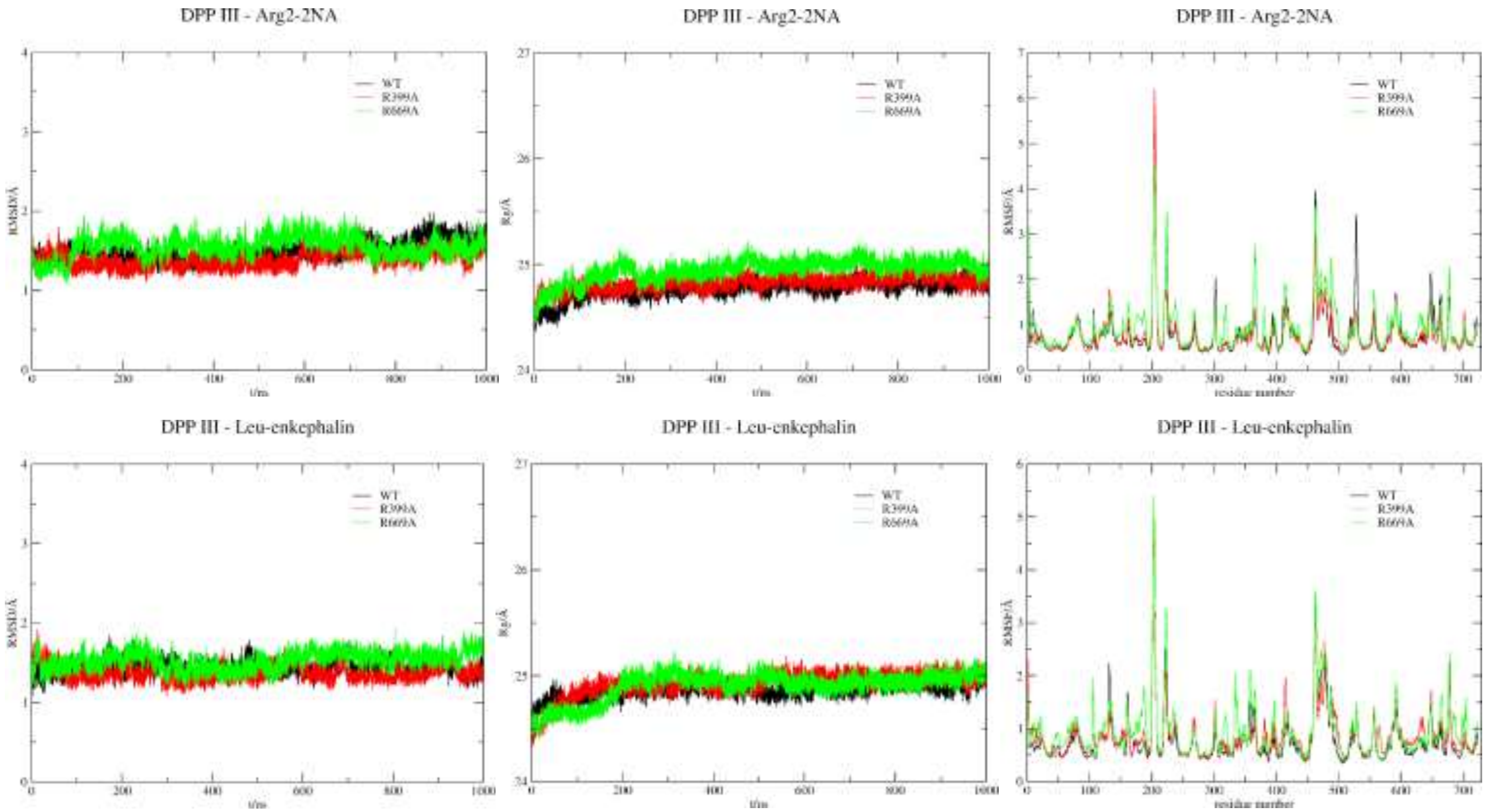
DPP III - Arg2-2NA
WT
R399A
R669A
DPP III - Arg2-2NA
WT
R399A
R669A
DPP III - Arg2-2NA
WT
R399A
R669A
residue number
DPP III - Leu-enkephalin
WT
R399A
R669A
DPP III - Leu-enkephalin
WT
R399A
R669A
DPP III - Leu-enkephalin
WT
R399A
R669A
residue number

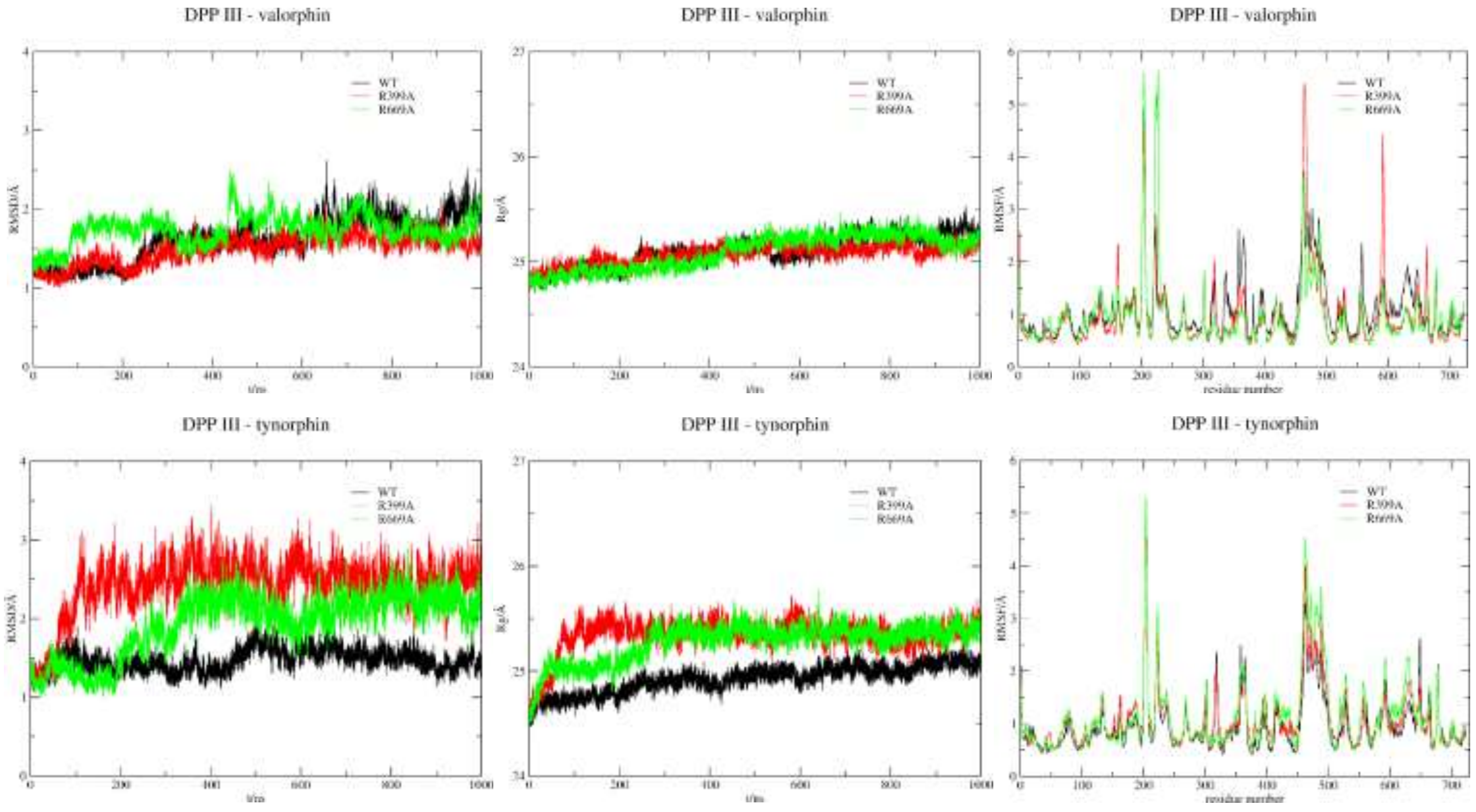

Figure S2. Profiles of root mean square deviations (RMSD), radius of gyration (Rg), and root mean square fluctuations (RMSF) from 1 μs long MD simulations of neuropeptides in complex with DPP III using the optimized complex structure as a reference. Enzyme backbone atoms (Cα, C, and N) were considered in the analysis. RMSD and Rg profiles were calculated excluding atoms from the flexible loop (residues 463-489).

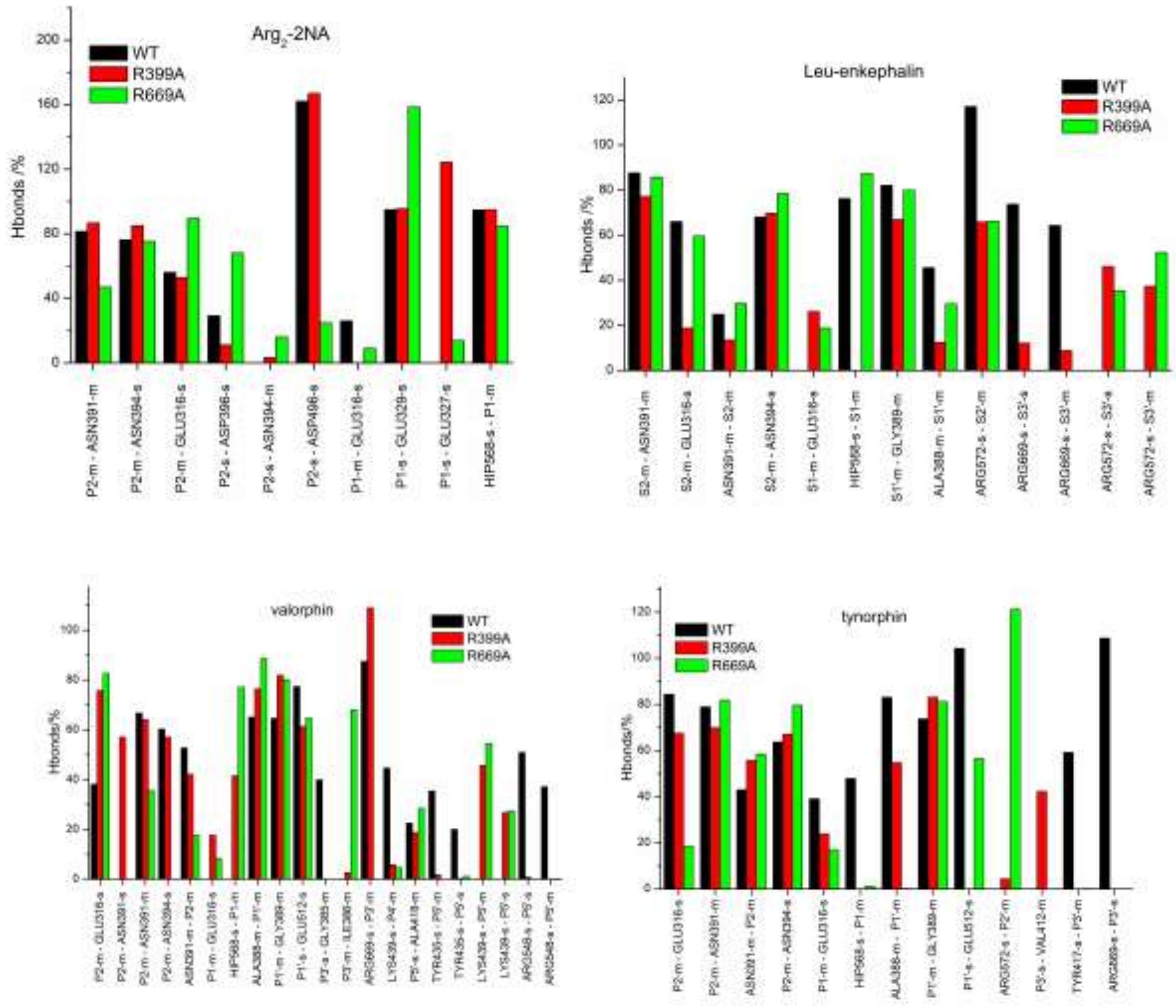

Figure S3. Occupancy of hydrogen bonds (shown only when occupancy is at least 20% in a complex) calculated with the Hbonds plugin (VMD) between the ligand and the rest of the protein. The angular cutoff was 45° and the distance cutoff was 3 Å. The labels on the X-axes indicate the interacting pair: the protein amino acid and the ligand subsite. If the name of the amino acid is on the left, it is involved as a hydrogen bond donor; if it is on the right, it is involved as a hydrogen bond acceptor. It is also indicated whether the amino acid interacts with the ligand *via* the main chain atoms (m) or the side chain atoms (s).

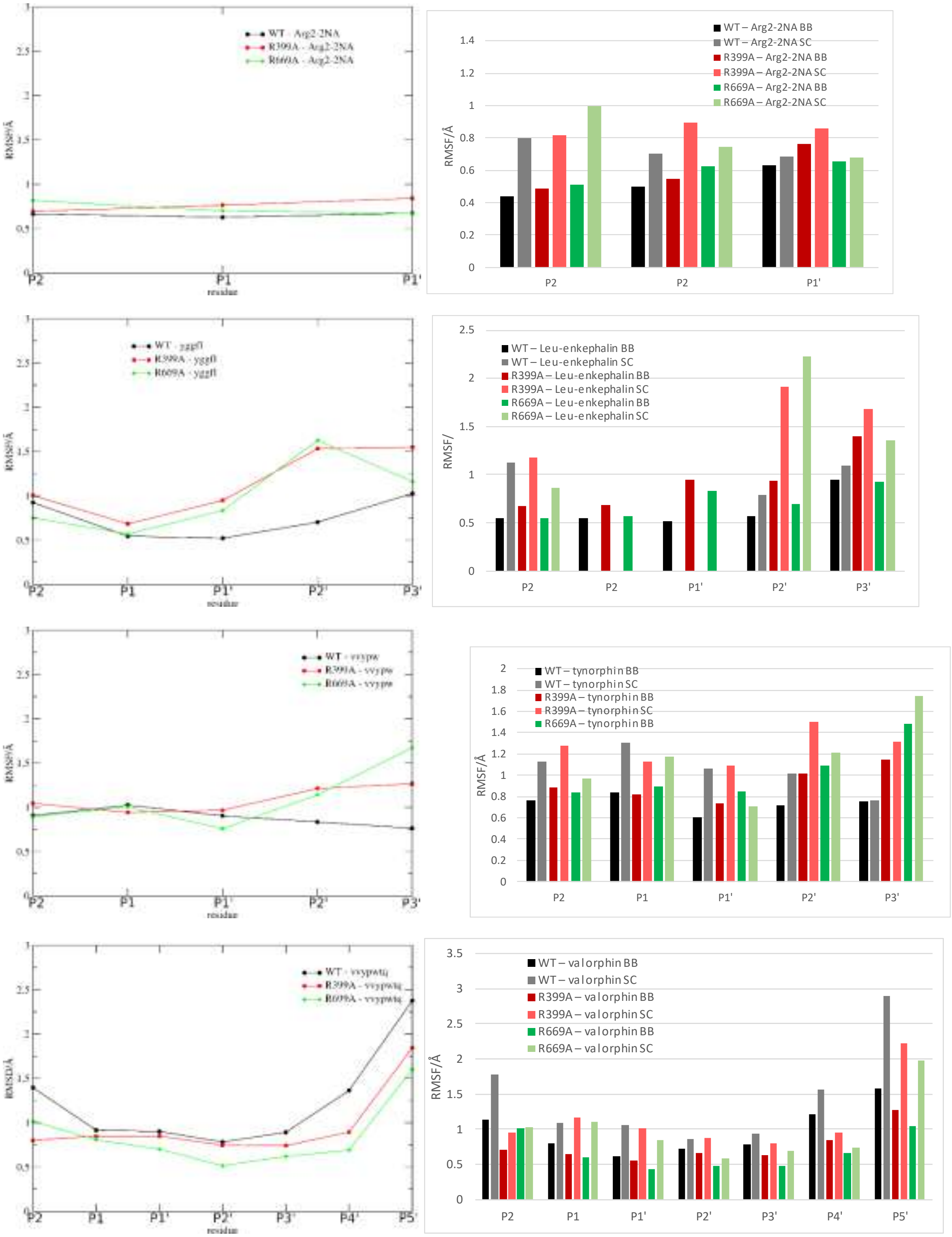

Figure S4. Left, RMSF values per ligand residue (labelled from P2 to P1'/P3'/P5' from N- to C-terminus, and calculated considering only the heavy atoms) obtained from 1 μs long MD simulations of different ligands in complex with DPP III. Right: RMSF values calculated separately for backbone (BB; considering Cα, C, N, and O atoms) and side chain (SC; heavy atoms) atoms.

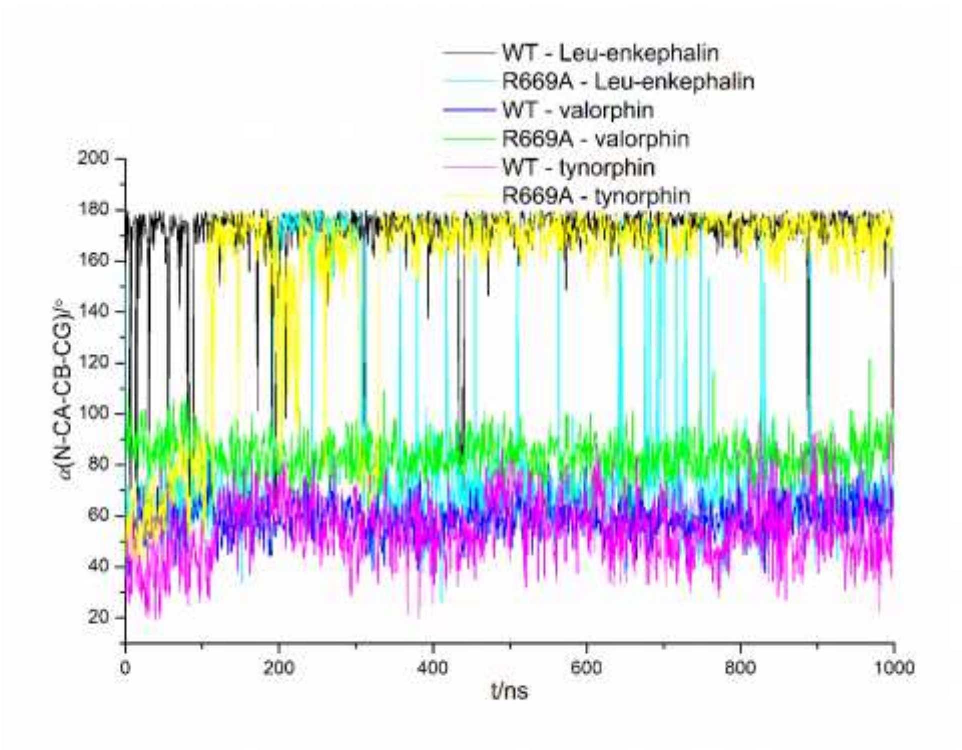

Figure S5. Change in the N-Cα-Cβ-Cγ dihedral angle of the ligand residue at the P3’ position during 1 μs long MD simulations.

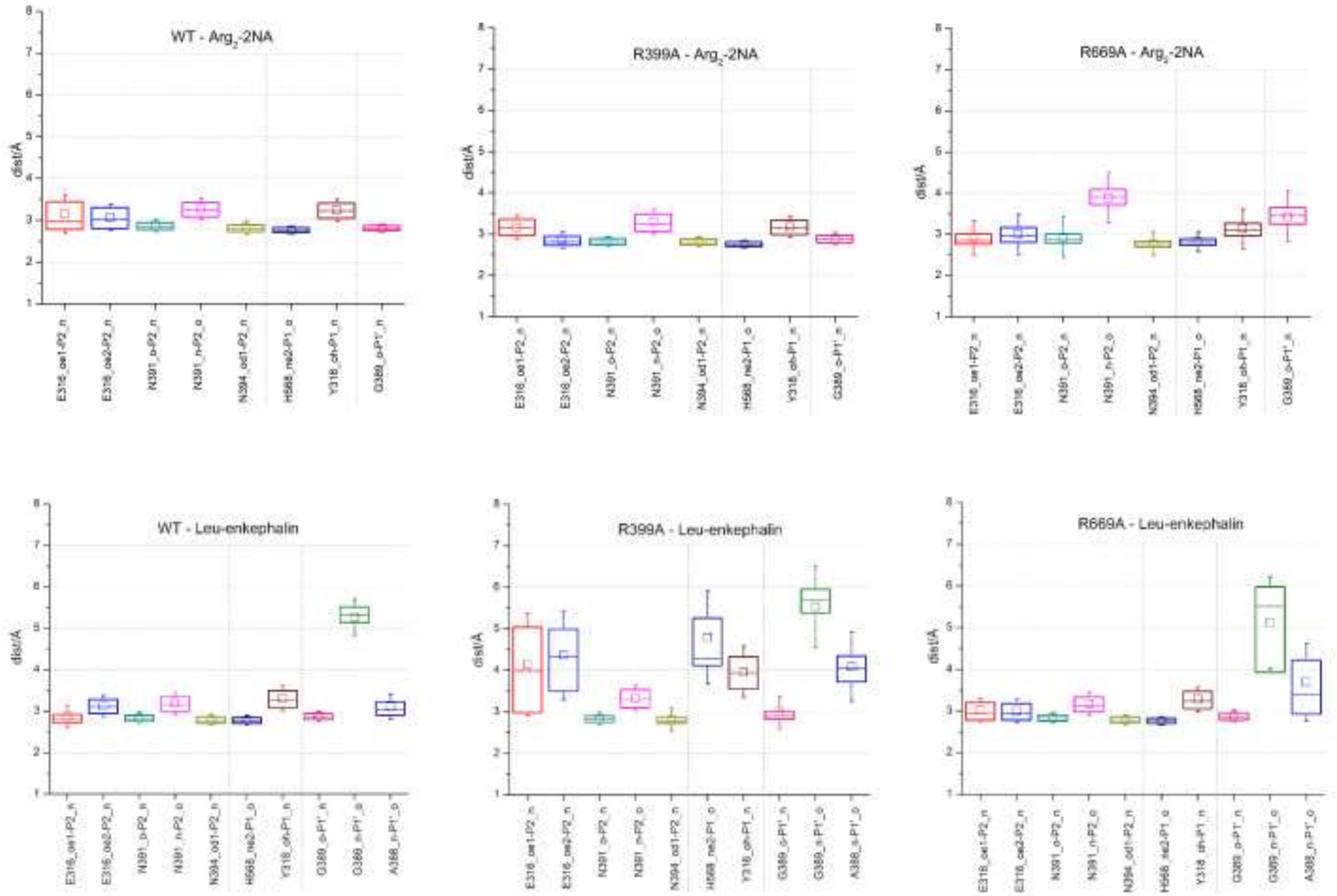
WT - Arg$_2$-2NA
R399A - Arg$_2$-2NA
R669A - Arg$_2$-2NA
WT - Leu-enkephalin
R399A - Leu-enkephalin
R669A - Leu-enkephalin
dist/Å

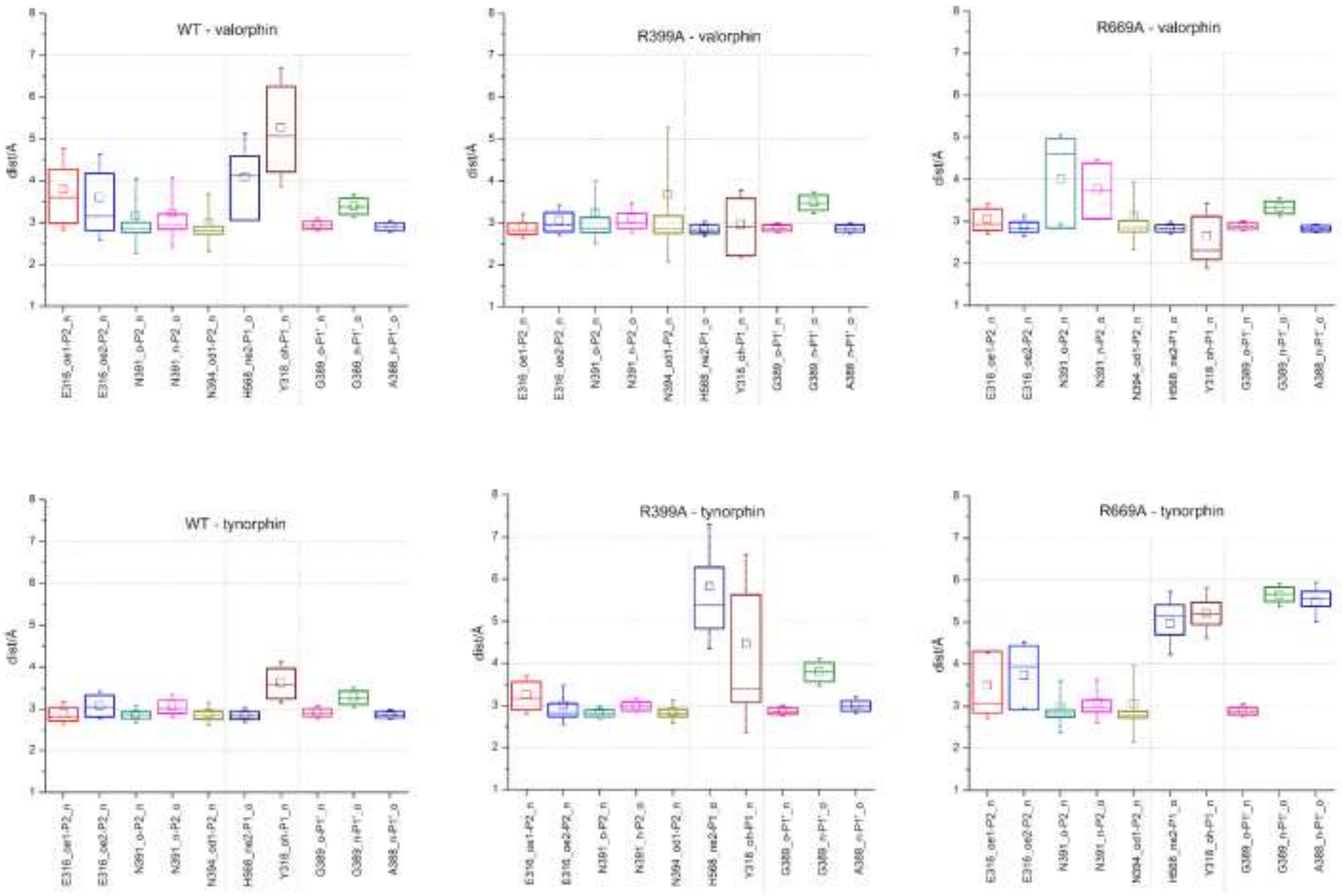

Figure S6. Box plots of the distances that keep ligand bound antiparallel to a β-strand of the lower protein domain and anchor its N-terminus (A389-N391, N394 and E316) and those that stabilize the ligand during hydrolysis (H568 and Y318). The amino acid residues of the peptide ligand are designated P1 to Pn and P1′ to Pn′, counting from the scissile peptide bond to the N- and C-termini of the peptides, respectively. The atoms between which the distance was calculated are indicated after the underscore.

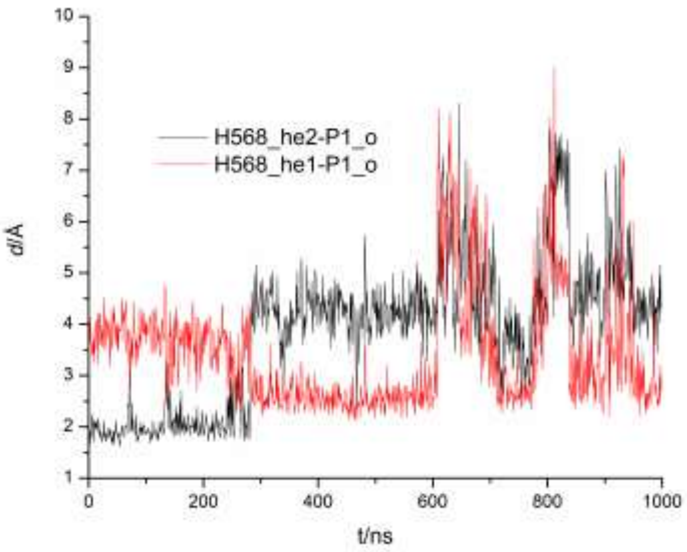

Figure S7. Change in distance between H568 (Nε-H) (black) and H568 (Cε-H) (red) and the carbonyl oxygen of the residue at the P1 position of the ligand during WT - valorphin MD simulations.

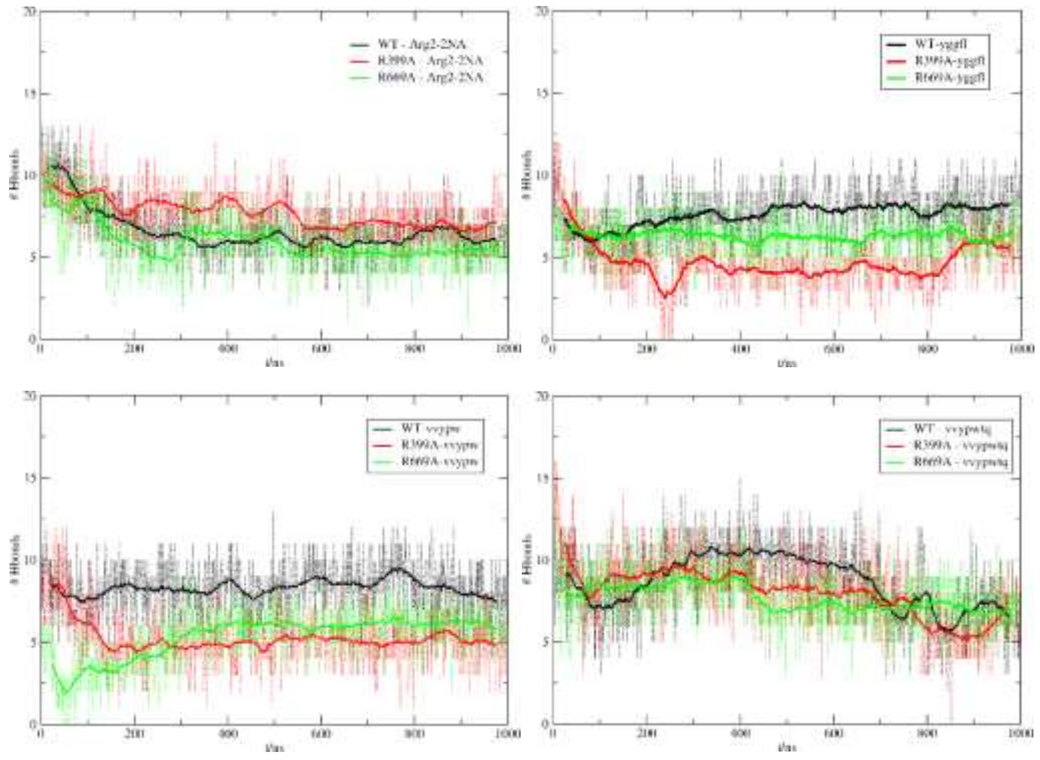

Figure S8. Number of hydrogen bonds (Hbonds) between the ligand and the rest of the protein calculated with the Hbond plugin in VMD. Cutoff values for angle and distance were 45° and 3 Å, respectively.

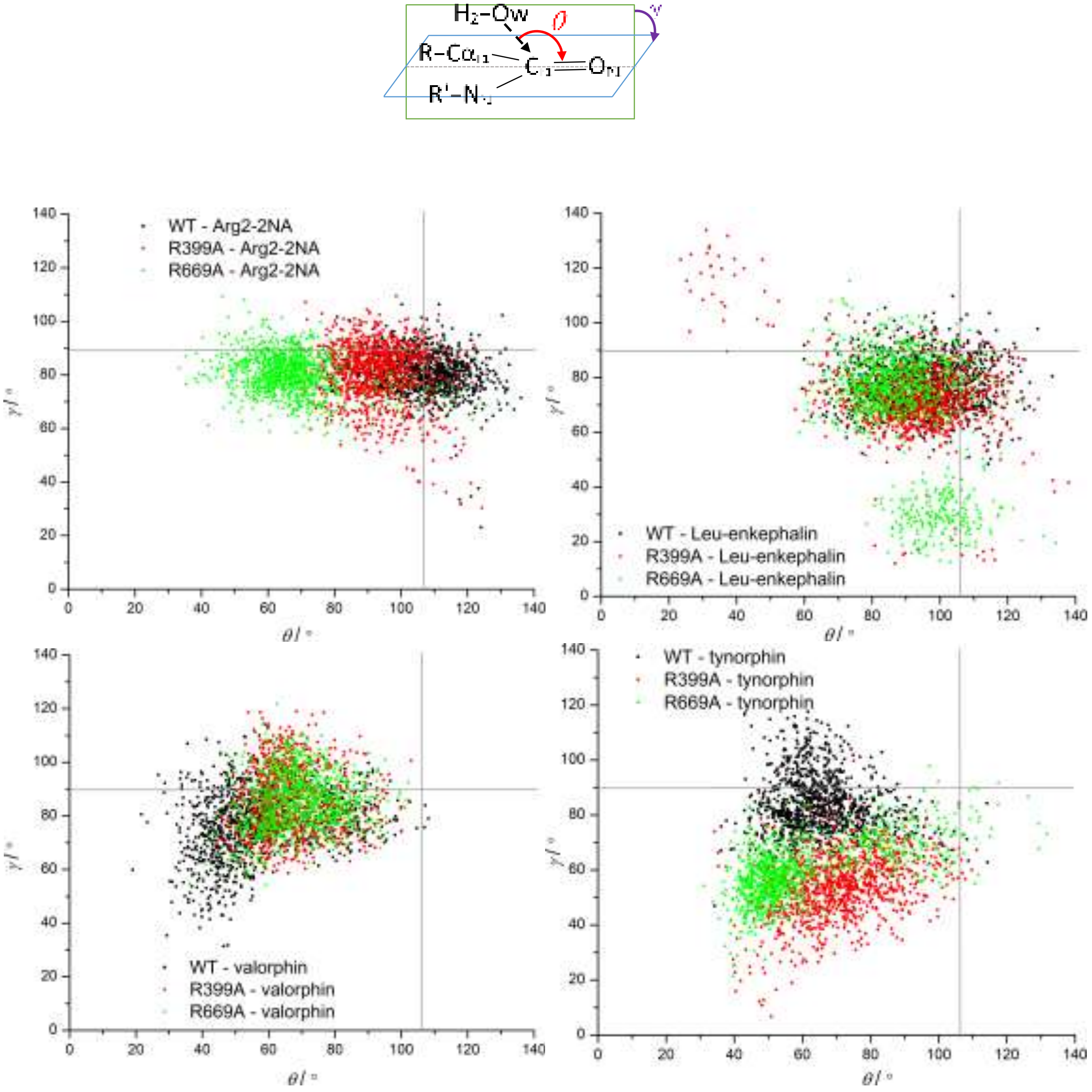

Figure S9. 2D diagrams of the: O-C-Ow angle (θ) defining the direction of OH− attack, and N-O-C-Ow dihedral angle (γ) which defines the angle between the plane of the 2nd peptide bond and the plane defined by the O-C-Ow atoms, more precisely, it indicates whether OH− attacks the carbonyl carbon atom from the direction perpendicular to the plane of the 2nd peptide bond (γ=90°) or from the side. N is the peptide amide nitrogen atom at the P1' position, O is the peptide carbonyl oxygen atom at the P1 position, C is the carbonyl carbon atom at the P1 position, and Ow is the oxygen atom of the activated water molecule. The gray lines indicate the Bürgi–Dunitz attack angle of θ=107°, and γ angle of 90°. The angles were calculated from 1 μs long MD simulations of WT (wild type) and mutated DPP III in complexes with neuropeptides.

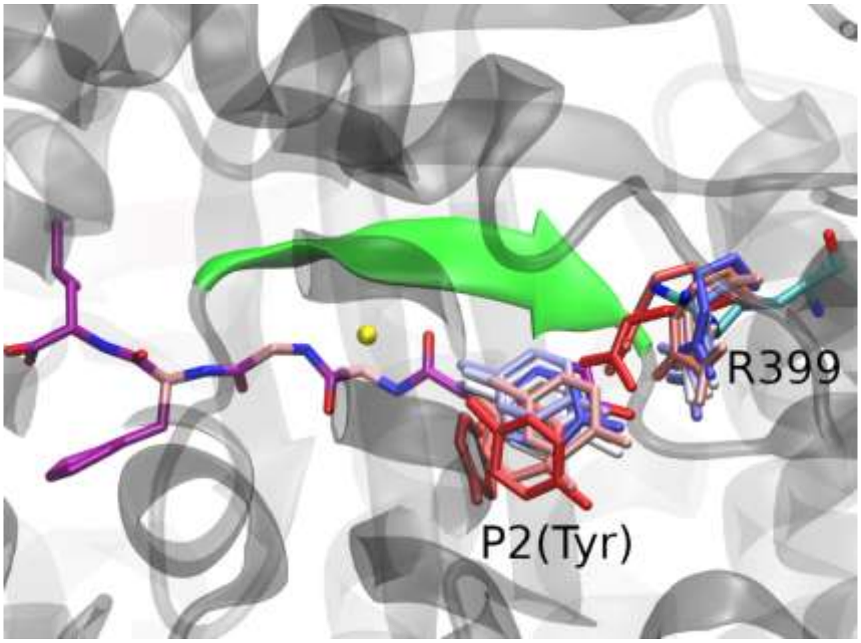

Figure S10. Wild type DPP III in complex with Leu-enkephalin (carbon atoms coloured magenta and light pink) in the structure obtained after 1 µs of MD simulations. The backbone of R399 (the position obtained after 1µs is shown with the carbon atoms in cyan) and the poses of tyrosine at position P2, which are sampled every 100 ns, are shown as sticks and coloured according to their position in the trajectory (the beginning of the trajectory in red, the middle in white, and the end in blue). The zinc ion is shown as a yellow sphere and the beta-sheet from the protein lower domain, involved in the ligand binding is coloured green. Hydrogens are omitted.

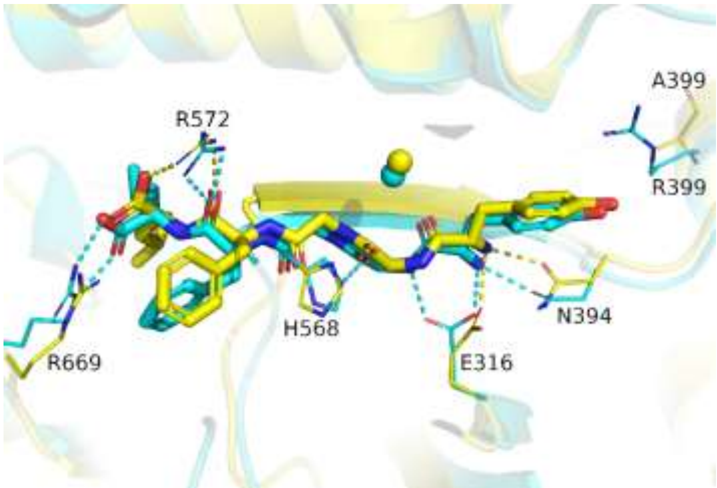

Figure S11. Binding of Leu-enkephalin in the wild type (cyan) and the R399A mutated (yellow) DPP III enzyme. Shown are optimized structures obtained after 1 µs of MD simulations. The amino acid residues that form hydrogen bonds (indicated by dashed lines) with the ligand are shown as thin sticks as are the amino acid residues that form polar interactions with the ligand. The β-sheet (residues A388-N391) from the lower protein domain involved in antiparallel binding of the ligand is shown as an opaque cartoon. The zinc ion is shown as a sphere. The hydrogen atoms are not shown, nor are the main chain atoms, except at A399.
